# Overexpression or Deletion of Ergosterol Biosynthesis Genes Alters Doubling Time, Response to Stress Agents, and Drug Susceptibility in *Saccharomyces cerevisiae*

**DOI:** 10.1128/mBio.01291-18

**Published:** 2018-07-24

**Authors:** Somanon Bhattacharya, Brooke D. Esquivel, Theodore C. White

**Affiliations:** a School of the Biological Sciences, University of Missouri, Kansas City, Kansas City, Missouri, USA; University of Texas Health Science Center

**Keywords:** *Saccharomyces cerevisiae*, antifungal drug resistance, ergosterol biosynthesis, ergosterol gene overexpression, ergosterol regulation, stress agents

## Abstract

Ergosterol (ERG) is a critical sterol in the cell membranes of fungi, and its biosynthesis is tightly regulated by 25 known enzymes along the ERG production pathway. The effects of changes in expression of each ERG biosynthesis enzyme in Saccharomyces cerevisiae were analyzed by the use of gene deletion or plasmid-borne overexpression constructs. The strains overexpressing the ERG pathway genes were examined for changes in doubling time and responses to a variety of stress agents. In addition, ERG gene overexpression strains and ERG gene deletion strains were tested for alterations in antifungal drug susceptibility. The data show that disruptions in ergosterol biosynthesis regulation can affect a diverse set of cellular processes and can cause numerous phenotypic effects. Some of the phenotypes observed include dramatic increases in doubling times, respiratory deficiencies on glycerol media, cell wall insufficiencies on Congo red media, and disrupted ion homeostasis under iron or calcium starvation conditions. Overexpression or deletion of specific enzymes in the ERG pathway causes altered susceptibilities to a variety of classes of antifungal ergosterol inhibitors, including fluconazole, fenpropimorph, lovastatin, nystatin, amphotericin B, and terbinafine. This analysis of the effect of perturbations to the ERG pathway caused by systematic overexpression of each of the ERG pathway genes contributes significantly to the understanding of the ergosterol biosynthetic pathway and its relationship to stress response and basic biological processes. The data indicate that precise regulation of ERG genes is essential for cellular homeostasis and identify several ERG genes that could be exploited in future antifungal development efforts.

## IMPORTANCE

A common target of antifungal drug treatment is the fungal ergosterol biosynthesis pathway. This report helps to identify ergosterol biosynthesis enzymes that have not previously been appreciated as drug targets. The effects of overexpression of each of the 25 ERG genes in S. cerevisiae were analyzed in the presence of six stress agents that target essential cellular processes (cell wall biosynthesis, protein translation, respiration, osmotic/ionic stress, and iron and calcium homeostasis), as well as six antifungal inhibitors that target ergosterol biosynthesis. The importance of identifying cell perturbations caused by gene overexpression or deletion is emphasized by the prevalence of gene expression alterations in many pathogenic and drug-resistant clinical isolates. Genes whose altered expression causes the most extensive phenotypic alterations in the presence of stressors or inhibitors have the potential to be drug targets.

## INTRODUCTION

Ergosterol (ERG) is the major sterol present in plasma and mitochondrial membranes of fungi and functions to maintain membrane fluidity, permeability, and structure (1). In addition, cell membranes contain microdomains called lipid rafts, which are formed by association of sterols and sphingolipids and are enriched with many biologically important molecules such as efflux pumps, sodium and potassium pumps, receptors, and nutrient transporters (1, 2). These microdomains are central to a variety of cellular processes, stress responses, and adaptations to the environment; maintaining lipid rafts is critical for proper functioning of the cells (1).

**FIG 1:**
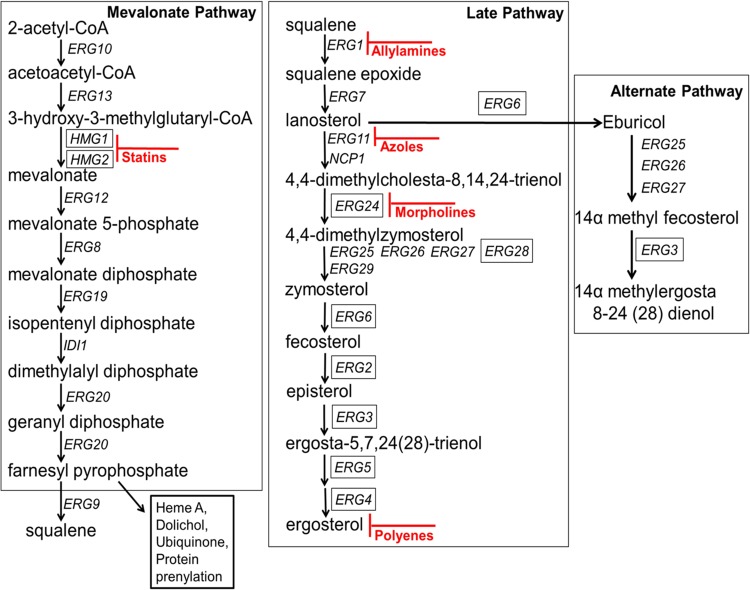
Ergosterol biosynthetic pathway. The box on the left diagrams the mevalonate pathway, which can channel products into different biosynthetic pathways. The box in the middle represents the late ergosterol pathway terminating in ergosterol. The box on the right represents an alternate pathway leading to the toxic fungistatic sterol [14α methylergosta 8-24-28 dienol]. Boxed gene names denote nonessential genes. Red names represent antifungal drugs and their targets of inhibition.

Sterol biosynthesis occurs in the endoplasmic reticulum (ER) and involves a cascade of 25 biosynthetic enzymes (Fig. 1). These enzymes are regulated in part by the zinc-cysteine finger transcription factor paralogs Upc2p/Ecm22p in Saccharomyces cerevisiae and by Upc2p in the pathogenic fungus Candida albicans (3, 4). This transcription factor acts as a sensor for cellular sterol levels and activates genes for sterol uptake and biosynthesis when sterol levels are reduced (4, 5).

The sterol biosynthetic pathway can be divided into the three parts: the "mevalonate" pathway, the "late" pathway, and the "alternate" pathway (Fig. 1). The mevalonate pathway synthesizes farnesyl pyrophosphate (FPP) from acetyl-coenzyme A (acetyl-CoA). FPP is an important intermediate in the biosynthesis of ubiquinone, dolichol, heme, sterols, and prenylated proteins (6). These products can be channeled into many other cellular pathways (6). Paralogs Hmg1p and Hmg2p (synthetic lethals) catalyze the third step (the rate-limiting step) in the mevalonate pathway (7). The remaining enzymes in the mevalonate section of the pathway are essential genes.

The late pathway is responsible for synthesizing ergosterol from FPP (Fig. 1). Erg1p and Erg11p represent two rate-limiting steps in this part of the pathway (7). Erg11p is a lanosterol 14-α-demethylase and functions in an association with Ncp1p. The coregulation of Erg11p and Ncp1p may contribute to the regulation of the entire ergosterol pathway (8). There are seven nonessential genes in the late pathway (indicated with boxed gene names in Fig. 1).

A branch from the late pathway is activated when Erg11p is inhibited under conditions such as by treatment with azoles (Fig. 1). We have designated this sequence of enzymatic reactions the "alternate pathway," in which, instead of proceeding toward the production of ergosterol, sterol intermediates are forced to reroute away from Erg11p. A by-product from this alternate pathway is a sterol metabolite dienol that is fungistatic to the cell (Fig. 1) (9). The shift from the late pathway to the alternate pathway has been shown to be mediated by *ERG6*, and the final step in the formation of the toxic 14α methylergosta 8-24 (22) dienol (referred to as "dienol") is catalyzed by *ERG3* (Fig. 1) (10). A combinatorial disruption in both *ERG11* and *ERG3* has been shown to circumvent the buildup of the dienol and leads to the development of resistance to azoles (9, 10).

Ergosterol is not present in mammalian cells, which instead produce cholesterol. This distinction makes fungal ergosterol and the ergosterol biosynthesis pathway successful targets of antifungal drugs for treatment of fungal infections in humans, animals, and plants. Classes of drugs targeting the ergosterol biosynthesis pathway are listed in Fig. 1 and include drugs that target the three rate-limiting enzymes Hmg1p, Erg1p, and Erg11p.

The statins, such as lovastatin (LOV), target human or fungal Hmg1p and are commonly used in humans to lower cholesterol levels. Allylamines, including terbinafine (TRB), target Erg1p and are effective against dermatophyte infections. Azoles, such as fluconazole (FLC), target Erg11p and are the most common antifungal drugs used to treat fungal infections (11, 12). Apart from these, several other drugs target different parts of the ergosterol pathway. Morpholines such as fenpropimorph (FEN), amorolfine (AMO), and tridemorph (TRI) target Erg24p (13). And finally, the polyenes amphotericin B (AMB) and nystatin (NYS) bind to ergosterol in the fungal membrane (14).

In pathogenic fungi, the mechanisms of drug resistance often include overexpression of membrane transporters, including ATP binding cassette transporters (ABC-T) and major facilitator superfamily transporters (MFS-T), which often show increased expression and efflux activities in resistant isolates (15-18). Additionally, azole resistance is sometimes correlated with alterations in ergosterol biosynthesis such as overexpression or point mutations in *ERG11*, the target of azoles (17, 18). Further, mutations in *ERG2*, *ERG3*, and *ERG6* that lead to incorporation of modified sterols into the membrane have also been characterized in drug-resistant clinical isolates of *Candida* spp. that exhibited cross-resistance to both azoles and AMB (9, 19, 20). As mentioned above with the alternate pathway, combinations of mutated genes of the ERG pathway can also lead to altered azole susceptibilities (9, 10).

The ergosterol pathway includes the following 9 nonessential genes: *HMG1*, *HMG2*, *ERG2*, *ERG3*, *ERG4*, *ERG5*, *ERG6*, *ERG24*, and *ERG28* (Fig. 1). Deletion strains for each of the 9 nonessential genes are viable but show disruption of ergosterol biosynthesis and accumulation of aberrant sterols leading to susceptibility to stress agents and osmotic/ionic stress, as well as abnormal calcium homeostasis and reduced efflux pump activities (1, 21-23). In one study, ERG pathway gene deletion strains exhibited abnormal mitochondrial structure and respiratory incompetence (24). While many ERG gene deletions increase susceptibility to stress agents, some deletion strains are resistant to medically important antifungals. For example, Δ*erg3* and Δ*erg6* strains are resistant to FLC and the polyenes AMB and NYS (1) (see Table S1 in the supplemental material).

10.1128/mBio.01291-18.4 TABLE S1  Fold change in drug susceptibilities caused by overexpression or deletion of *ERG* genes. Download TABLE S1, DOCX file, 0.01 MB. Copyright © 2018 Bhattacharya et al. 2018 Bhattacharya et al. This content is distributed under the terms of the Creative Commons Attribution 4.0 International license.

In this work, the phenotypic effects of altered expression of each of the key ergosterol genes were investigated in S. cerevisiae by gene deletion or by the use of plasmid-borne overexpression constructs. Overexpression enabled the characterization of gain-of-function phenotypes and of defects associated with misregulation of genes, identification of potential enzymatic bottlenecks, and recognition of genes that are more sensitive to perturbations in regulation. For each ERG gene overexpression strain, we measured cell doubling time, iron and calcium homeostasis, osmotic/ionic stress tolerance, respiration, cell wall biosynthesis, and protein translation inhibition and found that several cellular processes are affected by overexpression of specific ERG genes. The response to antifungal drug treatment was also measured for each of the *ERG* overexpression strains as well as the viable *ERG* gene deletion strains, and there were significant changes in antifungal drug susceptibilities caused by alterations in *ERG* gene expression.

## RESULTS AND DISCUSSION

### Expression analysis of plasmid-borne genes.

Quantitative reverse transcription-PCR (qRT-PCR) was used to analyze the mRNA expression levels for each *ERG* gene after induction in galactose-containing media (Gal media) as described in Text S1 in the supplemental material. All plasmid-borne *ERG* genes were found to be significantly (>2-fold) overexpressed in the presence of galactose compared to the corresponding endogenous *ERG* gene expression in the wild-type (WT) strain (see Fig. S1 in the supplemental material).

10.1128/mBio.01291-18.1 TEXT S1  qRT measurements of transcript levels. Download TEXT S1, DOCX file, 0.02 MB. Copyright © 2018 Bhattacharya et al. 2018 Bhattacharya et al. This content is distributed under the terms of the Creative Commons Attribution 4.0 International license.

10.1128/mBio.01291-18.3 FIG S1  Induced expression levels of plasmid-borne *ERG* genes. The mRNA transcript levels of the plasmid-borne genes were analyzed using qRT-PCR after galactose induction. The horizontal line shows significant 2-fold overexpression compared to W303-1A (WT). The genes are listed in their order along the ergosterol biosynthesis pathway. Download FIG S1, TIF file, 0.2 MB. Copyright © 2018 Bhattacharya et al. 2018 Bhattacharya et al. This content is distributed under the terms of the Creative Commons Attribution 4.0 International license.

### Analysis of growth phenotypes.

The doubling time of strain W303-1A expressing each of the 25 plasmid-borne *ERG* genes was analyzed as the strains grew in either noninducing glucose-containing media (Glu media) or Gal media for 96 h.

**FIG 2:**
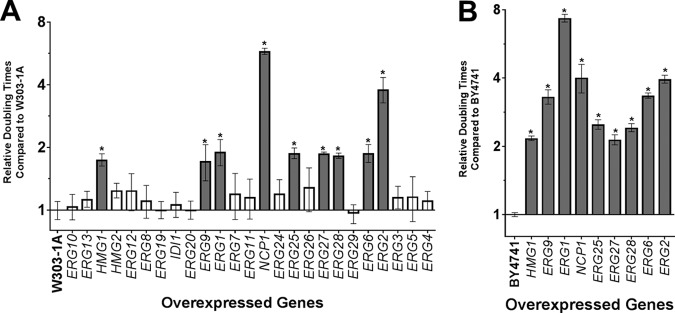
Doubling times of strains overexpressing ERG genes. Strains carrying plasmids containing ERG genes were grown in Gal media to induce gene overexpression (A) in the W303-1a strain background and (B) in the BY4741 strain background. Significant values are plotted as gray bars. The genes are listed in their order along the ergosterol biosynthesis pathway.

In Gal media, 9 of 25 strains overexpressing *ERG* genes showed significantly increased doubling time compared to the W303-1A WT strain (Fig. 2A; see also Table 1). A longer doubling time corresponds to slower growth. The slow growing strains included those overexpressing four nonessential genes (*HMG1*, *ERG2*, *ERG6*, and *ERG28*) and five essential genes (*ERG1*, *ERG9*, *ERG25*, *ERG27*, and *NCP1*). To confirm that the significantly slower growth was not a by-product of the W303-1A background strain, the plasmids containing the *HMG1*, *ERG1*, *ERG2*, *ERG6*, *ERG9*, *ERG25*, *ERG27*, *ERG28*, and *NCP1* genes were also overexpressed in another S. cerevisiae WT strain, BY4741, a derivative of S288C. The slow-growth phenotype caused by overexpression of these 9 genes was confirmed in BY4741 (Fig. 2B). Among the slow-growing strains, the strains overexpressing *ERG1* (BY4741 background) and *NCP1* (W303-1A background) had the longest doubling times. The doubling time of S. cerevisiae WT strain BY4741 in Glu media was 3.5 h and in Gal media was 4.5 h. The doubling time of S. cerevisiae WT strain W303-1A in Glu media was 3.0 h and in Gal media was 4.5 h.

**TABLE 1:** Summary of stress agent effects on overexpression strains

Gene name	Essential gene?	Growth rate	No. of stress agents	Stress agent^a^						
				Fe^+2^	Ca^+2^	NaCl	GLY	CR	CHX	SDS
*ERG10*	Yes		1			x				
*ERG13*	Yes		3		x	x		x		
*HMG1*	No	Low	5	x	x	x	x	x		
*HMG2*	No		4		x	x	x	x		
*ERG12*	Yes		2		x			x		
*ERG8*	Yes		1		x					
*ERG19*	Yes		1			x				
*IDI1*	Yes		1		x					
*ERG20*	Yes		2		x	x				
*ERG9*	Yes	Low	3	x	x	x				
*ERG1*	Yes	Low	3	x	x	x				
*ERG7*	Yes		3		x	x		x		
*ERG11*	Yes		3		x	x	*	x		
*NCP1*	Yes	Low	5	x	x	x		x	x	
*ERG24*	No		3		x	x			x	
*ERG25*	Yes	Low	3	x	x	x				
*ERG26*	Yes		1			x				
*ERG27*	Yes	Low	3	x	x	x				
*ERG28*	No	Low	5	x	x	x	x	x		
*ERG29*	Yes		1		x					
*ERG6*	No	Low	6	x	x	x	x	x	x	
*ERG2*	No	Low	5	x	x		x	x	x	
*ERG3*	No		1		x					
*ERG5*	No		2		x	x				
*ERG4*	No		2		x	x				
										
Total		9		9	22	19	5	10	4	0

a GLY, glycerol; *, possible improved growth under that condition; x, slow growth under that condition.

The slow growth of these strains could have been caused by the accumulation of sterol intermediates or by disruption of an enzyme-specific downstream effect(s) on ergosterol biosynthesis. For example, overexpression of *ERG6* could encourage the initiation of the alternate pathway (Fig. 1), resulting in the accumulation of the dienol, known to be toxic to the cell (25).

*HMG1* is a rate-limiting, feedback-sensitive enzyme in the early steps of the ERG pathway. Overexpression of this tightly regulated enzyme is thought to cause dramatic negative-feedback downregulation of ERG pathway genes leading to an accumulation of presqualene intermediates, reduced cellular ergosterol, and slow growth (26).

Several proteins are part of a multienzyme complex encoded by *ERG25*, *ERG26*, *ERG27*, and *ERG28*. Overexpression of *ERG25*, *ERG27*, or *ERG28* (Fig. 1) could disrupt the normal stoichiometry of the complex, leading to deviations or disruptions in the sequential catalytic reactions, or could have other deleterious effects on cell growth, although overexpression of *ERG26* does not have this effect. The complex consisting of *ERG25*, *ERG26*, *ERG27*, and *ERG28* is additionally thought to interact directly with *ERG6* (27), whose overexpression also causes slow growth (Fig. 2).

### Requirements for iron.

**FIG 3:**
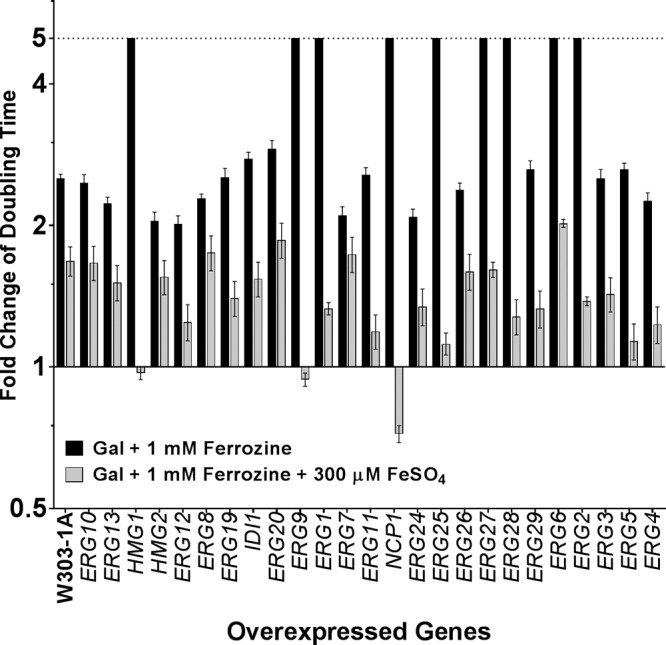
Iron requirement of strains overexpressing ERG genes. The doubling time of strains overexpressing each of the ERG genes was measured in Gal media alone, in Gal media plus 1 mM ferrozine (black bars), and in Gal media plus 1 mM ferrozine plus 300 µM FeSO_4_ (gray bars). The genes are listed in their order along the ergosterol biosynthesis pathway.

Iron deprivation initiated by the presence of ferrozine, an iron chelating agent, caused slower growth for every strain tested, while additional specific growth inhibition was observed for some overexpression strains (Fig. 3).

There was complete inhibition of growth observed in 9 strains overexpressing the genes *HMG1*, *ERG1*, *ERG2*, *ERG6*, *ERG9*, *ERG25*, *ERG27*, *ERG28*, and *NCP1* in the presence of ferrozine (Fig. 3; see also Table 1). These 9 strains are the same strains that exhibited slow growth in Gal media compared to the results seen with the WT strain (strains highlighted in gray in Fig. 2). Strains exhibiting the slow-growth phenotype were clearly already metabolically stressed by *ERG* gene overexpression, and this effect was exacerbated by the additional metabolic stress of iron starvation. Growth was at least partially or completely restored to these slow-growing strains when FeSO_4_ was added to the Gal media containing ferrozine (Fig. 3).

Since iron is a cofactor for many enzymes (28, 29), iron deprivation or alterations in iron homeostasis can affect sterol biosynthesis and many other enzymatic functions (30). Forced overexpression of iron-requiring enzymes such as those involved in sterol biosynthesis could exacerbate this disruption in cellular iron stores and lead to slow growth. Interestingly, there was a difference between the strains overexpressing the isoenzymes *HMG1* and *HMG2* with respect to the effects of iron starvation. The difference in response could be related to specialization of the enzymes for aerobic or anaerobic environments; *HMG1* encodes the predominant enzyme under aerobic conditions whereas *HMG2* expression is induced under anaerobic conditions (30, 31). This may explain why the *HMG1*-overexpressing strain is not as well equipped as the *HMG2*-overexpressing strain to grow under conditions of iron starvation, which mimic low-oxygen conditions (31).

### Requirements for calcium.

**FIG 4:**
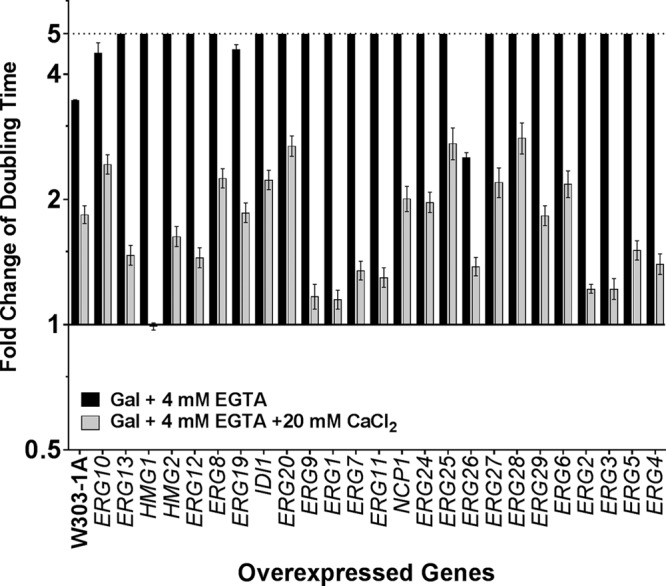
Calcium requirement of strains overexpressing ERG genes. The doubling time of strains overexpressing each of the ERG genes was measured in Gal media alone, in Gal media plus 4 mM EGTA (black bars), and in Gal media plus 4 mM EGTA plus 20 mM CaCl_2_ (gray bars). The genes are listed in their order along the ergosterol biosynthesis pathway.

Calcium deprivation caused by the presence of the calcium chelating agent EGTA had a significant growth-inhibitory effect on the majority of the *ERG*-overexpressing strains (Fig. 4). The growth of 22 (*HMG1*, *HMG2*, *ERG1*, *ERG2*, *ERG3*, *ERG4*, *ERG5*, *ERG6*, *ERG7*, *ERG9*, *ERG11*, *ERG12*, *ERG13*, *ERG20*, *ERG24*, *ERG25*, *ERG27*, *ERG28*, *ERG29*, *NCP1*, and *IDI1*) of the 25 strains was completely inhibited compared to that seen with the WT strain under low-calcium conditions, as indicated in Fig. 4 (see also Table 1).

Many calcium transporters and signaling molecules are localized in lipid rafts on the cell membrane (32). These microdomains made up of sterols and sphingolipids may be altered in cells with disrupted ergosterol regulation. Sensitivity to calcium starvation in a majority of the ERG-overexpressing strains could be the result of defective calcium regulation, including import, export, and signaling, which may be lethal to cells.

Additionally, a previous study indicated that an excess of calcium ions in culture provided a protective effect and was able to positively modulate fungal responses to stressors, mutations, or inhibitors (33). Thus, calcium starvation may have the opposite effect and may magnify growth defects in strains with disrupted ERG biosynthesis.

### Tolerance of hyperosmotic or ionic stress.

**FIG 5:**
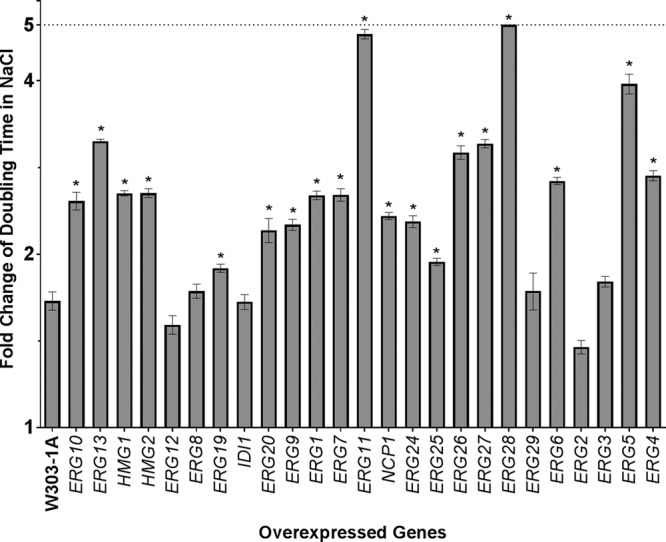
Cellular response to osmotic/ionic stress. The doubling time of strains overexpressing each of the ERG genes was measured in Gal media alone or in Gal media with 1.2 M NaCl. The genes are listed in their order along the ergosterol biosynthesis pathway.

Osmotic/ionic stress resulting from a high NaCl concentration caused significantly slower growth in 19 (*HMG1*, *HMG2*, *ERG1*, *ERG4*, *ERG5*, *ERG6*, *ERG7*, *ERG9*, *ERG10*, *ERG11*, *ERG13*, *ERG19*, *ERG20*, *ERG24*, *ERG25*, *ERG26*, *ERG27*, *ERG28*, and *NCP1*) of the 25 strains compared to the WT strain (Fig. 5; see also Table 1).

Previous research has highlighted the complex cellular adjustments required to adapt to osmotic and ionic stress, with particular importance attributed to the transcriptional regulation of ergosterol biosynthesis (34). In wild-type cells, hyperosmotic stress causes ergosterol production to be repressed and the regulatory system consisting of membrane channels and water and solute sensors works to keep the cells in ionic/osmotic balance (35). Forced overexpression of the individual ERG genes and changes to the membrane composition may be counterproductive for the cells with respect to overcoming salt stress, accounting for the reduced growth in 19 of the ERG strains.

### Utilization of a nonfermentable carbon source.

**FIG 6:**
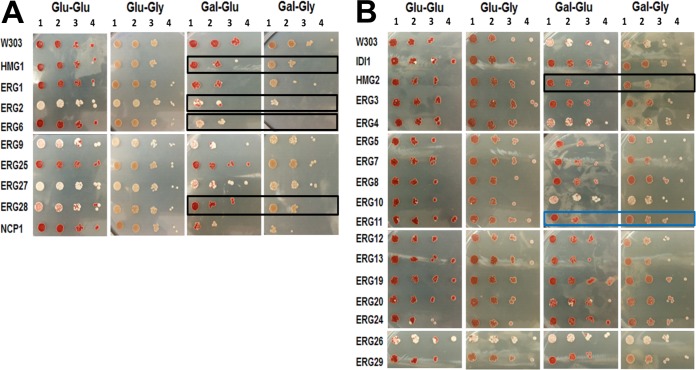
Utilization of a nonfermentable carbon source to elucidate respiratory deficiencies. Slow growers (A) and normal growers (B) were grown in Glu media for 24 h and then plated on Glu media (Glu-Glu) or Gly media (Glu-Gly). Strains were also grown in Gal media for 48 h and then plated on Glu media (Gal-Glu) or Gly media (Gal-Gly). The black outlines highlight the strains with a growth deficiency on glycerol media. Blue outlines highlight growth improvement on glycerol media.

Since ergosterol is a component of many cellular membranes, including the mitochondrial membrane, overexpression of ergosterol pathway genes could disrupt or otherwise affect mitochondrial function. *ERG*-overexpressing strains were analyzed for respiratory deficiency on glycerol media (Fig. 6; see also Table 1). Panel A of Fig. 6 includes the 9 strains that had showed a slow-growth phenotype under conditions of growth in galactose. Panel B includes the strains that showed a normal doubling time in galactose.

While plating on Glu or Gly media was no longer an inducing condition, the effects of ERG gene overexpression from inducing media were still present as indicated by the differences in growth levels between Glu-Glu and Gal-Glu media, in which the Gal phenotype persisted even after plating on noninducing media. Poor growth on glycerol or other nonfermentable carbon sources indicates respiratory incompetence.

There were no significant growth deficiencies in any of the strains in comparisons of the Glu-Glu spots to Glu-Gly spots under control conditions, indicating the absence of respiratory deficiencies when plasmid expression is not strongly induced. Under inducing conditions, the strains overexpressing *HMG1*, *ERG2*, *ERG6*, *ERG28*, and *HMG2* showed a significant growth deficiency on Gly media (Fig. 6). This indicates that respiratory deficiency was present in these strains when the gene was overexpressed.

It is also possible that the overexpression of certain genes along the ERG pathway could actually improve respiration and mitochondrial function. The strain overexpressing *ERG11* showed increased growth on glycerol media (Gal-Gly) compared to glucose media (Gal-Glu) (Fig. 6B).

### Response to the cell wall stress.

**FIG 7:**
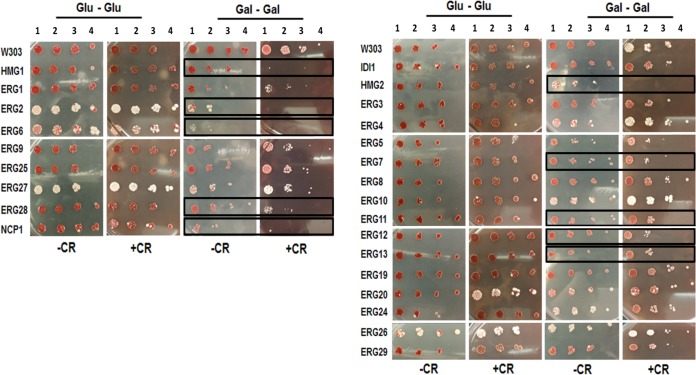
Response to cell wall stress. Slow growers (A) and normal growers (B) were grown in Glu media and plated on Glu media (Glu-Glu) in the absence (−CR) or presence (+CR) of 64 µg/ml of Congo red. The strains were also grown in Gal media and plated on Gal media (Gal-Gal) in the absence (−CR) or presence (+CR) of 64 µg/ml of Congo red. The black outlines highlight strains sensitive to CR.

Perturbations in ergosterol production and the yeast cell membrane can directly affect the macromolecular structure and composition of the cell wall by disruption of the membrane-associated proteins that build and shape the cell wall and extracellular matrices (36). In addition, ER stress caused by accumulation of misfolded proteins has been associated with a decline of cell wall integrity (37). Congo red (CR) was used to analyze cell wall stability and to determine if overexpression of *ERG* genes disrupted the cell wall integrity (Fig. 7; see also Table 1). Panel A of Fig. 7 includes the 9 strains that had a slow-growth phenotype under conditions of growth in galactose. Panel B includes the strains that had a normal doubling time in galactose. Under Gal-inducing conditions (Gal-Gal), the strains overexpressing *HMG1*, *ERG2*, *ERG6*, *ERG28*, *NCP1*, *HMG2*, *ERG7*, *ERG11*, *ERG12*, and *ERG13* showed a significant growth deficiency on media containing CR (Fig. 7).

**FIG 8:**
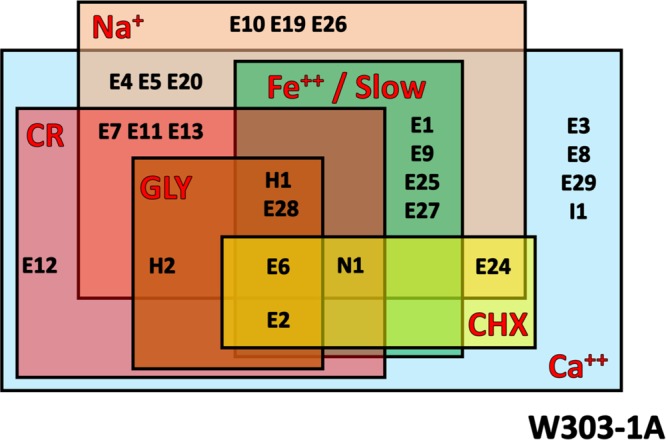
Summary of stress agent phenotypes. The strains whose designations appear in squares demonstrated a phenotype that was altered from the WT phenotype under that condition. "E" signifies an *ERG* gene, "I1" signifies the *IDI1* gene, "H" signifies the *HMG* genes, and "N1" signifies the *NCP1* gene.

Table 1 and Fig. 8 summarize the effects of *ERG* gene overexpression on doubling time and on susceptibility to six stress agents. Note that overexpression of *ERG2* and *ERG6* had an effect on susceptibility to all six stress agents, while all 25 genes had an effect on susceptibility to at least one agent.

### Susceptibility to antifungal agents—minimum inhibitory concentration.

**FIG 9:**
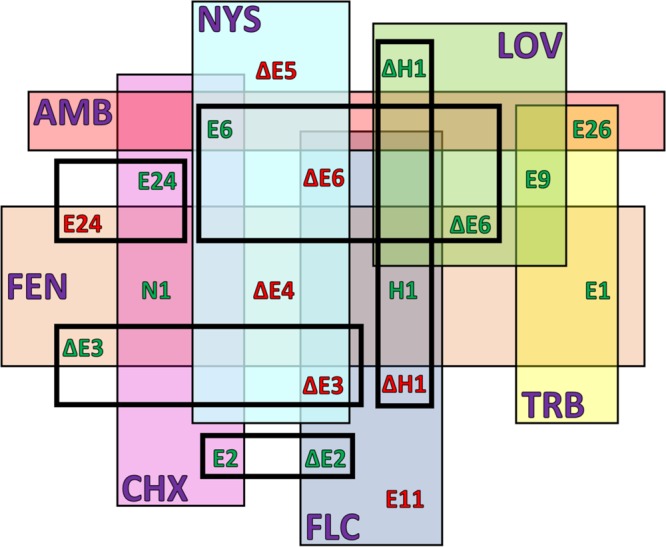
Summary of antifungal drug susceptibilities. The strains whose designations appear in squares demonstrated drug susceptibility that was altered from that seen with the WT strain for that drug. The letter E signifies *ERG* genes, the letter H signifies the *HMG* genes, and "N1" signifies the *NCP1* gene. Δ = gene deletion. Drug abbreviations are indicated with purple text. Green text represents increased susceptibility. Red text represents increased resistance. The presence of identical genes is indicated with boxes with thick borders. The different drugs are indicated with boxes with thin borders.

The strains were analyzed for changes in drug susceptibility caused by overexpression or deletion of ergosterol pathway genes (Fig. 9) (Table 2; see also Table S1 and S2 in the supplemental material). Overexpression of the 25 plasmid-borne *ERG* genes was analyzed in the wild-type W303-1A strain. Drug susceptibility was also analyzed in 9 strains with deletion of nonessential *ERG* genes (Δ*hmg1*, Δ*hmg2*, Δ*erg24*, Δ*erg28*, Δ*erg6*, Δ*erg2*, Δ*erg3*, Δ*erg5*, and Δ*erg4*) in the wild-type BY4741 strain. The MIC of each drug (lovastatin [LOV], terbinafine [TRB], fluconazole [FLC], fenpropimorph [FEN], nystatin [NYS], amphotericin B [AMB], sodium dodecyl sulfate [SDS], and cycloheximide [CHX]) was determined for all strains in both Glu media (data not shown) and Gal media. The values listed in Table S1 indicate the fold change in the MIC for that strain compared to the MIC for the WT strain.

**TABLE 2:** Strains with significant changes in drug susceptibility

Drug	Target(s)	S. cerevisiae strain(s) with increased^a^:		Total no. of strains
		Susceptibility	Resistance	
LOV	*HMG1*, *HMG2*	Δ*hmg1*, *ERG9*, Δ*erg6*	None	3
TRB	*ERG1*	*ERG9*, *ERG1*, *ERG26*	None	3
FLC	*ERG11*	*HMG1*, Δ*erg2*	Δ*hmg1*, *ERG11*, Δ*erg6*, Δ*erg3*	6
FEN	*ERG24*	*HMG1*, *ERG1*, *NCP1*, Δ*erg6*, Δ*erg3*	*ERG24*, Δ*erg4*	7
NYS	Ergosterol	*ERG6*	Δ*erg6*, Δ*erg3*, Δ*erg5*, Δ*erg4*	5
AMB	Ergosterol	*ERG26*, *ERG6*	None	2
SDS	Cell membrane	None	None	0
CHX	Protein translation	*NCP1*, *ERG24*, *ERG6*, *ERG2*	None	4

a An uppercase Greek delta (Δ) denotes a strain with a gene deletion(s). All other gene designations denote gene overexpression in Gal media.

10.1128/mBio.01291-18.5 TABLE S2  Complementation of deletion strains with plasmid-borne genes. Download TABLE S2, DOCX file, 0.01 MB. Copyright © 2018 Bhattacharya et al. 2018 Bhattacharya et al. This content is distributed under the terms of the Creative Commons Attribution 4.0 International license.

Table 2 and Fig. 9 summarize the results of the MIC analysis. For all drugs associated with the ergosterol pathway, gene overexpression or deletion resulted in a change in drug susceptibility. For the known ergosterol inhibitors LOV, TRB, FLC, and FEN, the target gene was not the only gene to show an effect.

Hypersusceptibility to LOV was observed in the strain overexpressing *ERG9*. Overexpression of *ERG9* and the possible accumulation of squalene (Fig. 1) may induce transcriptional downregulation of Hmg1p/Hmg2p, which have negative feedback that is sensitive to the accumulation of sterol intermediates and thus could account for the LOV susceptibility in this strain (26).

The Δ*hmg1* strain was hypersusceptible to LOV, and overexpression of *HMG1* complemented the phenotype (Table S2). However, the Δ*hmg2* strain showed a LOV MIC equal to that seen with wild-type strain. A previous study demonstrated that Hmg1p is the predominant isoenzyme and is responsible for 84% of the enzyme activity (38). Thus, the effects of LOV on the Δ*hmg2* strain may have been masked by the presence of a WT *HMG1* copy in this strain. LOV hypersusceptibility was also observed in the Δ*erg6* strain, which grew slowly, possibly affecting LOV MICs.

The allylamine terbinafine is thought to target Erg1p, which represents a rate-limiting step in the late pathway (Fig. 1). The strains overexpressing *ERG1* and *ERG9* were hypersusceptible to TRB. Erg9p converts FPP to squalene, a substrate for Erg1p, and so the overexpression of Erg9p or Erg1p may bias FPP toward ergosterol biosynthesis and away from its other cellular roles, such as those represented by heme, dolichol, and ubiquinone, as well as protein prenylation, causing susceptibility in these strains (Fig. 1). TRB hypersusceptibility was also observed in the strain overexpressing *ERG26*, which is part of the *ERG25*, *ERG*26, *ERG27*, and *ERG28* complex. None of the deletion strains were significantly affected by TRB compared to the WT strain results.

The primary target of azoles such as fluconazole is Erg11p, and, as expected, the strain overexpressing *ERG11* showed significantly increased resistance to FLC. Overexpression of *ERG11* was previously shown to increase cellular ergosterol content (39). Hypersusceptibility to FLC was observed in the strain overexpressing *HMG1*, which agrees with a previous observation indicating that overexpression of *HMG1* results in decreased synthesis of ergosterol (26). The decrease in cellular ergosterol content associated with overexpression of *HMG1* could account for the hypersensitivity to FLC. Conversely, the Δ*hmg1* strain was resistant to FLC, possibly as a consequence of a reverse effect, in which cellular ergosterol synthesis was stimulated in the absence of *HMG1*.

FLC resistance was observed in the Δ*erg3* and Δ*erg6* deletion strains. Strains Δ*erg3* and Δ*erg6* were unable to synthesize the toxic dienol that accumulated in the WT cell upon azole exposure (Fig. 1) and hence are FLC resistant. The Δ*erg2* strain was hypersusceptible to FLC.

### AMB and NYS.

The medically important polyenes AMB and NYS target ergosterol in the fungal membrane. Strains with reduced ergosterol content or altered membrane sterol composition have been shown previously to have polyene resistance (40). The strain overexpressing *ERG6* is hypersusceptible to both polyenes. A recent study demonstrated that strains overexpressing *ERG6* have increased sterol levels (41), which would account for the susceptibility to these inhibitors.

The strain overexpressing *ERG26*, upstream of *ERG6*, was hypersusceptible to AMB but was not hypersusceptible to NYS. In addition, the Δ*erg3*, Δe*rg4*, Δ*erg5*, and Δ*erg6* deletion strains were resistant to NYS and yet showed nearly wild-type AMB MICs. While AMB and NYS both target ergosterol in the membrane, their modes of action or targets of recognition may be slightly different as illustrated by the differences between the polyene effects on these strains (Table S1).

### CHX and SDS.

CHX targets ribosomal protein translation, potentially causing abnormal protein synthesis. SDS can destabilize the plasma membrane, affecting cell growth and viability (42). CHX hypersusceptibility was observed in four strains (*ERG24*, *ERG2*, *ERG6*, and *NCP1*). However, none of the 25 strains were affected by SDS (Table 1).

### FEN.

Morpholines such as fenpropimorph are ERG pathway inhibitors thought to target Erg24p. Seven strains with an *ERG* gene deletion or overexpression had significantly altered FEN MICs. Observation of FEN resistance in the *ERG24* overexpressing strain provides support for the idea that Erg24 is the main morpholine drug target. The Δ*erg4* strain was also resistant to FEN.

The strains overexpressing *HMG1*, *ERG1*, and *NCP1* were hypersusceptible to FEN. *HMG1* and *ERG1* are checkpoint genes in the ERG pathway, while *NCP1* is a cofactor for *ERG11*. FEN hypersusceptibility was also observed in the Δ*erg3* and Δ*erg6* deletion strains.

There was great variability between the ERG overexpressor strains in response to stressors and inhibitors, highlighting the unique specialization of each enzyme and the pleiotropic nature of ergosterol biosynthesis disruption. Data from the strains affected by the greatest number of stressors and inhibitors could indicate the sections of the pathways that are most sensitive to disruption in regulation and that thus have the most potential to be used as drug targets. Strains overexpressing genes *ERG2*, *ERG6*, *ERG28*, *HMG1*, and *NCP1* were affected by five or more stress agents and also showed slower growth. While *HMG1*, *ERG1*, *ERG11*, and *ERG24* are the targets for current antifungal drugs, these data suggest that the *ERG2*, *ERG6*, *ERG28*, and *NCP1* genes could also be considered potential new drug targets. Additionally, the strains that overexpress *ERG9*, *ERG1*, *ERG25*, and *ERG27* are affected by three stress agents and have a slow-growth phenotype. Clearly, misregulation of these genes has far-reaching effects on the cell.

While we have described an initial investigation of the phenotypic results of deregulation of the ergosterol pathway, a more comprehensive analysis of each strain could ultimately bring to light mechanistic explanations for each of the phenotypes. Further work would include sterol analysis, including analysis of total cell sterol content and of sterol intermediates, and analysis of changes in plasma membrane composition for each ERG overexpression strain. Differences in gene expression and proteomics profiles between the strains would also provide insight into the mechanisms of the observed phenotypes associated with this essential and dynamic pathway.

## MATERIALS AND METHODS

### Yeast strains.

S. cerevisiae strain W303-1A (*MAT***a** *leu2-3*,*112 trp1-1 can1-100 ura3-1 ade2-1 his3-11*,*15*) was used for most of the phenotypic experiments. S. cerevisiae strain BY4741 and ERG gene mutants Δ*hmg1*, Δ*hmg2*, Δ*erg2*, Δ*erg3*, Δ*erg4*, Δ*erg5*, Δ*erg6*, Δ*erg24*, and Δ*erg28* were obtained from the S. cerevisiae deletion library for strain BY4741 (*MAT***a** *leu2v0 his3v1 ura3v0 met15v0*) (7). Plasmid complementation experiments described in Text S2 in the supplemental material were performed using the deletion library mutants.

10.1128/mBio.01291-18.2 TEXT S2  Complementation analysis of plasmid-borne genes. Download TEXT S2, DOCX file, 0.01 MB. Copyright © 2018 Bhattacharya et al. 2018 Bhattacharya et al. This content is distributed under the terms of the Creative Commons Attribution 4.0 International license.

### Plasmid construction.

Genomic DNA from the S. cerevisiae W303-1A strain was isolated as described previously (43). Each ERG gene was PCR amplified and verified for the correct gene size on a 0.8% agarose gel. Oligonucleotides used for amplification are listed in Table S3 in the supplemental material.

10.1128/mBio.01291-18.6 TABLE S3  Oligonucleotides used for ERG amplification from genomic DNA. Download TABLE S3, DOCX file, 0.01 MB. Copyright © 2018 Bhattacharya et al. 2018 Bhattacharya et al. This content is distributed under the terms of the Creative Commons Attribution 4.0 International license.

Forward oligonucleotides included a (5′) 30-bp sequence with homology to the galactose-regulated *GAL1* promoter (*GAL1-*p), while the reverse oligonucleotides had a (5′) 30-bp sequence with homology to the *CYC1* terminator (*CYC1-*t) for homologous recombination into the pYES2 plasmid. The plasmid was digested with PvuII, and each ERG gene was inserted. The pYES2 plasmid was a gift from the laboratory of A. Idnurm (University of Missouri—Kansas City [UMKC]).

Yeast cells were transformed using the lithium-acetate method (44), and successful transformants were selected on CSM-ura agar plates (1.7 g of yeast nitrogen base without ammonium sulfate and without amino acids, 5 g/liter of ammonium sulfate, 0.8 g/liter of CSM-ura [complete supplementation mixture with uracil] powder, 20 g/liter agar, 2% glucose). Plasmids were isolated, used for transformation of Escherichia coli (TOP10 competent cells; Sigma-Aldrich) (45), and plated on Luria-Bertani agar containing 100 µg/ml of ampicillin. The plasmids were then isolated from E. coli, all 25 plasmid-borne ERG genes were sequenced using oligonucleotides listed in Table S4, and the sequences were compared with the sequences available in the Saccharomyces Genome Database (http://www.yeastgenome.org). The sequencing analysis confirmed that all ERG gene sequences matched the corresponding published sequences for those genes and that the plasmid orientations of the genes were correct.

10.1128/mBio.01291-18.7 TABLE S4  Oligonucleotides used for ERG gene sequencing and qRT-PCR. Download TABLE S4, DOCX file, 0.01 MB. Copyright © 2018 Bhattacharya et al. 2018 Bhattacharya et al. This content is distributed under the terms of the Creative Commons Attribution 4.0 International license.

### Strain growth conditions.

S. cerevisiae strain W303-1A was transformed with plasmids carrying the S. cerevisiae *ERG* genes, and transformants were selected at 30°C in CSM-ura. All strains were grown in CSM-ura plus 2% glucose (Glu media) for noninducing conditions. Gene expression was induced under the control of the Gal1 promoter, and overexpression was performed in CSM-ura plus 2% galactose (Gal media). CSM-ura plus 3% glycerol (Gly media) was used as a medium containing a nonfermentative carbon source in one experiment. All strains were stored in Glu media plus 30% glycerol at −80°C.

### Reagents.

Restriction enzymes used for constructing plasmids were obtained from Promega. Stress agents and drugs for susceptibility testing were obtained from Sigma-Aldrich, St. Louis, MO. These included AMB, CaCl_2_, CHX, CR, EGTA, ferrozine, FeSO_4_, FEN, FLC, LOV, NYS, SDS, and TRB. Unless otherwise indicated, all materials and plasticware were from Fisher Scientific.

### Calculation of doubling time.

Doubling times were analyzed for the strains expressing each of the 25 ERG genes in Glu media and Gal media. First, growth curves were generated for each strain by growing the strain in a 96-well plate (Costar 3699; Fisher Scientific) containing either Glu media or Gal media and incubation at 30°C with constant shaking for 96 h in a BioTek Synergy H1 plate reader (BioTek Inc., USA). The optical density at 600 nm (OD_600_) in each well was measured automatically every 15 min over 24 h for Glu media and over 48 h in Gal media.

Similar growth curve analyses were performed for the iron and calcium requirement analysis and the osmotic/ionic stress analysis. Strains were grown in Gal media containing 1 mM ferrozine, 1 mM ferrozine plus 300 µM FeSO_4_, 4 mM EGTA, 4 mM EGTA plus 20 mM CaCl_2_, and 1.2 M NaCl. The change in doubling time due to the stress agent was calculated as fold change, where the fold change value represents the doubling time of the individual strain in Gal media with the stress agent/the doubling time of the individual strain in Gal-only media. Strains with 5-fold change or higher (dotted line) were considered to have complete growth inhibition.

Doubling times were calculated using Graph Pad Prism 7.0. First, the midpoint of the exponential phase (the inflection point) of the growth was analyzed using the double derivative function. Second, the range to generate the doubling time was fixed as the period from 5 h before to 5 h after the inflection point for each growth curve.

### Glycerol spot assay.

Each strain was grown in Glu media and spotted on agar plates either with Glu media (Glu-Glu, denoting overnight growth in Glu and plating on Glu) or with Gly media (Glu-Gly, denoting overnight growth in Glu and plating on Gly). Similarly, each strain was grown in Gal media and spotted on agar plates with either Glu media (Gal-Glu, denoting overnight growth in Gal and plating on Glu) or Gly media (Gal-Gly, denoting overnight growth in Gal and plating on Gly). Glu-Glu, Glu-Gly, and Gal-Glu plates were the control plates used in this experiment.

Starting at an OD_600_ of 0.1, the strains were spotted in a total of four 10-fold serial dilutions in decreasing cell concentrations (shown from left to right in Fig. 6 for each condition tested). All plates were incubated for 96 h at 30°C and then imaged with a color digital camera. For this assay, a reduction in colony growth (compared to the control) at colony spot position 1, 2, or 3 on the plate was considered representative of a significant growth deficiency.

### Congo red spot assay.

Each strain was grown in Glu media and then spotted on Glu media (Glu-Glu, denoting overnight growth in Glu and plating on Glu) with or without Congo red at 64 µg/ml. Similarly, each strain was grown in Gal media and spotted on Gal media (Gal-Gal, denoting overnight growth in Gal and plating on Gal) with or without Congo red at 64 µg/ml. Plates with Glu-Glu without CR (−CR) and Gal-Gal without CR (−CR) were important control plates used in this experiment. The strains were spotted and analyzed as described above for the glycerol spot assay.

### Susceptibility testing.

Susceptibilities to the AMB, CHX, FLC, FEN, LOV, NYS, SDS, and TRB drugs were tested on the S. cerevisiae strains overexpressing ERG genes.

MIC analysis was based on the CLSI protocol with the following adjustments for the ERG overexpression strains. Each strain was grown in Glu media (repressing conditions) or in Gal media (inducing conditions) and was used to perform the MIC analysis. Cells were grown in 96-well plates containing a gradient of drug in a 2-fold serial dilution in the Glu media or the Gal media and incubated at 30°C with shaking for 48 h and 96 h, respectively. MIC plates were read by the use of a BioTek 96-well plate reader. All MIC analyses were performed in biological duplicate, and the values were averaged.

### Statistics.

The experiments were done in biological duplicate or triplicate, and the error bars represent standard errors. One-way analysis of variance (ANOVA) was performed with Holm Sidak’s multiple-comparison test (*, *P* < 0.05).
