# Supplementary material for: Overexpression or Deletion of Ergosterol Biosynthesis Genes Alters Doubling Time, Response to Stress Agents, and Drug Susceptibility in Saccharomyces cerevisiae
Source: mBio. 2018 Jul 24;9(4):e01291-18. doi: 10.1128/mBio.01291-18 (PMC6058291; doi:10.1128/mBio.01291-18)
Supplement: TABLE S1 [file mbo004183972st1.docx]

**Supplemental Table 1. Fold Change in Drug Susceptibilities Caused by Overexpression or Deletion of *ERG* genes**

| **Strains** | **LOV** | **TRB** | **FLC** | **FEN** | **NYS** | **AMB** | **SDS** | **CHX** |
| --- | --- | --- | --- | --- | --- | --- | --- | --- |
| **WT** | **1** | **1** | **1** | **1** | **1** | **1** | **1** | **1** |
| ***ERG10*** | **2** | **1** | **1** | **2** | **1** | **1** | **1** | **1** |
| ***ERG13*** | **2** | **2** | **2** | **1** | **1** | **1** | **1** | **1** |
| ***HMG1*** | **2** | **1** | **4** | **8** | **1** | **2** | **1** | **1** |
| ***∆hmg1*** | **4** | **1** | **4** | **2** | **2** | **1** | **1** | **1** |
| ***HMG2*** | **2** | **2** | **2** | **2** | **1** | **1** | **1** | **1** |
| ***∆hmg2*** | **1** | **1** | **2** | **1** | **2** | **1** | **1** | **1** |
| ***ERG12*** | **2** | **2** | **1** | **1** | **1** | **1** | **1** | **1** |
| ***ERG8*** | **1** | **1** | **1** | **1** | **1** | **1** | **1** | **1** |
| ***ERG19*** | **2** | **1** | **1** | **2** | **1** | **1** | **1** | **1** |
| ***IDI1*** | **1** | **1** | **1** | **1** | **1** | **1** | **1** | **1** |
| ***ERG20*** | **1** | **1** | **1** | **2** | **1** | **1** | **1** | **1** |
| ***ERG9*** | **4** | **4** | **2** | **1** | **1** | **1** | **1** | **1** |
| ***ERG1*** | **1** | **4** | **2** | **4** | **1** | **2** | **1** | **1** |
| ***ERG7*** | **1** | **1** | **2** | **1** | **1** | **2** | **1** | **1** |
| ***ERG11*** | **1** | **1** | **4** | **1** | **1** | **1** | **1** | **1** |
| ***NCP1*** | **2** | **2** | **1** | **16** | **1** | **1** | **1** | **4** |
| ***ERG24*** | **1** | **1** | **1** | **>8** | **1** | **2** | **1** | **4** |
| ***∆erg24*** | **1** | **2** | **1** | **1** | **1** | **1** | **1** | **1** |
| ***ERG25*** | **2** | **1** | **2** | **1** | **1** | **2** | **1** | **1** |
| ***ERG26*** | **1** | **4** | **1** | **1** | **1** | **4** | **1** | **1** |
| ***ERG27*** | **2** | **2** | **2** | **1** | **1** | **2** | **1** | **1** |
| ***ERG28*** | **2** | **1** | **2** | **2** | **1** | **2** | **1** | **1** |
| ***∆erg28*** | **1** | **1** | **1** | **2** | **1** | **1** | **1** | **1** |
| ***ERG29*** | **1** | **1** | **1** | **1** | **1** | **1** | **1** | **1** |
| ***ERG6*** | **1** | **2** | **1** | **1** | **4** | **200** | **1** | **4** |
| ***∆erg6*** | **4** | **2** | **4** | **>200** | **>8** | **2** | **1** | **1** |
| ***ERG2*** | **2** | **1** | **2** | **2** | **1** | **1** | **1** | **4** |
| ***∆erg2*** | **1** | **2** | **4** | **1** | **1** | **1** | **1** | **1** |
| ***ERG3*** | **1** | **2** | **2** | **2** | **1** | **2** | **1** | **1** |
| ***∆erg3*** | **1** | **1** | **8** | **8** | **4** | **2** | **1** | **1** |
| ***ERG5*** | **1** | **1** | **2** | **2** | **1** | **1** | **1** | **1** |
| ***∆erg5*** | **1** | **1** | **2** | **2** | **8** | **1** | **1** | **1** |
| ***ERG4*** | **2** | **2** | **1** | **2** | **1** | **1** | **1** | **1** |
| ***∆erg4*** | **1** | **2** | **2** | **>4** | **4** | **1** | **1** | **1** |

Yellow boxes represent slow-growing strains

Red box denotes significant resistance

Blue box denotes significant hyper-susceptibility

Strains that had at least 4 fold reduced susceptibility are colored dark red and strains that had at least 4 fold increased susceptibility are shaded dark blue. MIC differences that are two-fold difference from WT are indicated by a light red or light blue shading to identify potential patterns of resistance or susceptibility but are not considered significant at this time.
