# Supplementary material for: Overexpression or Deletion of Ergosterol Biosynthesis Genes Alters Doubling Time, Response to Stress Agents, and Drug Susceptibility in Saccharomyces cerevisiae
Source: mBio. 2018 Jul 24;9(4):e01291-18. doi: 10.1128/mBio.01291-18 (PMC6058291; doi:10.1128/mBio.01291-18)
Supplement: TEXT S1 [file mbo004183972s1.docx]

**Measurements of Transcript Levels:** Quantitative-reverse transcriptase PCR (qRT-PCR) was used to analyze the level of expression of the ergosterol genes in the presence of galactose. Three colonies of each strain were each inoculated in Gal-media and incubated at 30^o^ C with 180 rpm of shaking. Total RNA was extracted from exponentially growing cells in Gal-media using Qiagen RNeasy kit following the manufacturer’s guidelines. The concentration and purity of the extracted RNAs were analyzed using the Take3 nanodrop feature of the Biotek Synergy H1 plate reader (Biotek Instruments. Inc USA). An absorbance ratio (A260/A280) ≥ 2.0 was considered to be sufficiently pure RNA. Further, the RNA samples were analyzed on a 1.2% agarose gel and the appearance of two distinct ribosomal RNA bands signified RNA integrity. 1 µg of total RNA was treated with DNAseI (Thermo-scientific) following the manufacturer’s protocol. cDNA was prepared from DNAse-treated RNA using an Invitrogen cDNA kit with Superscript III Reverse Transcriptase following the manufacturer’s instructions.

qRT-PCR was performed in Applied Biosystem 7500 Real-Time PCR System using the oligonucleotides listed in Supplemental Table 4 These oligonucleotides were designed using Primer3 software, and all amplicon sizes are in the range of 150-250 bp. qRT-PCR was done using Maxima SYBR Green qPCR master mixes (Thermo-scientific) following the manufacturer’s protocol. qRT-analysis was performed in triplicate for each strain. The housekeeping genes actin (*ACT1*) and transcription elongation factor (*TEF3*) were used as internal controls. The data were normalized to the wild type W303-1A gene expression. Specifically, the fold change in the expression was calculated using 2^-ΔΔct^. First, the Ct values of each ERG gene were compared to the Ct values of *ACT1* gene in that strain. Next, the *ACT1*-normalized values of the genes were compared to the WT (W303-1A) *ACT1*-normalized values for those genes to calculate fold change. Error bars were calculated as previously described (Livak and Schmittgen, 2001). Fold changes for each *ACT1*-normalized gene was confirmed against *TEF3*-normalized gene expression. A 2-fold or greater change in expression from WT was considered significant.

Most of the plasmid-borne *ERG* genes had more than 10-fold overexpression compared to WT gene expression (*ERG10, ERG13, HMG1, HMG2, ERG12, ERG8, ERG19, ERG20, ERG9, ERG1, NCP1, ERG25, ERG27, ERG28, ERG29, ERG6, ERG2, ERG3, ERG5*), and some genes were even expressed at 100-fold greater than WT (*IDI1, ERG7, ERG11, ERG24, ERG26, ERG4*) (Fig S1).
