# Supplementary figures and images for: Overexpression or Deletion of Ergosterol Biosynthesis Genes Alters Doubling Time, Response to Stress Agents, and Drug Susceptibility in Saccharomyces cerevisiae
Source: mBio. 2018 Jul 24;9(4):e01291-18. doi: 10.1128/mBio.01291-18 (PMC6058291; doi:10.1128/mBio.01291-18)

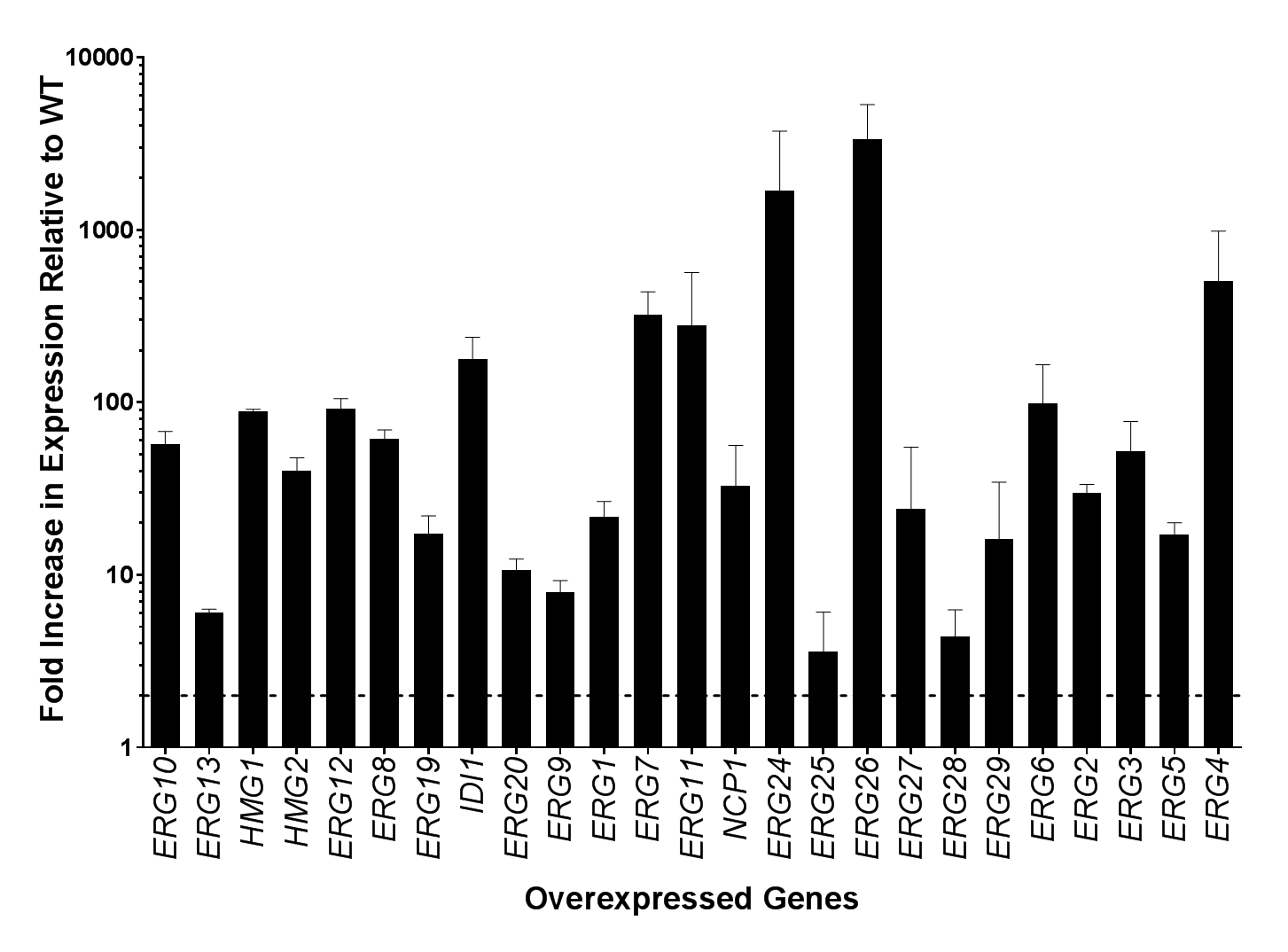

Supplement: FIG S1 [file mbo004183972sf1.tif]
