# Supplementary material for: Overexpression or Deletion of Ergosterol Biosynthesis Genes Alters Doubling Time, Response to Stress Agents, and Drug Susceptibility in Saccharomyces cerevisiae
Source: mBio. 2018 Jul 24;9(4):e01291-18. doi: 10.1128/mBio.01291-18 (PMC6058291; doi:10.1128/mBio.01291-18)
Supplement: TABLE S2 [file mbo004183972st2.docx]

| Supplemental Table 2. Complementation of Deletion Strains with Plasmid-Borne Genes | | |
| --- | --- | --- |
| Gene | **Deletion Phenotype** | **Complementation Phenotype** |
| *HMG1*^a^ | Susceptible to LOV,  Resistance to FLC | WT |
| *HMG2*^a^ | WT | plasmid compliments ∆*hmg1* |
| *ERG24*^b^ | WT | WT |
| *ERG28* | WT | WT |
| *ERG6* | Resistant to FLC, NYS,  Susceptible to FEN, LOV | WT |
| *ERG2* | Susceptible to FLC,  Slow-Growth | WT |
| *ERG3* | Resistant to FLC, NYS,  Susceptible to FEN | WT |
| *ERG5* | Resistant to NYS | WT |
| *ERG4* | Resistant to FEN, NYS | WT |

1. Synthetic lethal combination
2. Overexpression leads to FEN resistance in deletion strain
