# Supplementary material for: Overexpression or Deletion of Ergosterol Biosynthesis Genes Alters Doubling Time, Response to Stress Agents, and Drug Susceptibility in Saccharomyces cerevisiae
Source: mBio. 2018 Jul 24;9(4):e01291-18. doi: 10.1128/mBio.01291-18 (PMC6058291; doi:10.1128/mBio.01291-18)
Supplement: TEXT S2 [file mbo004183972s2.docx]

**Complementation Analysis of Plasmid-Borne Genes**

In addition to sequencing and qRT expression analysis, complementation analyses were performed for the nine non-essential *ERG* genes to verify their functionality and ability to complement the endogenous gene as shown in Supplemental Table 1. The activities of the nine non-essential ERG enzymes were analyzed for phenotypic complementation when expressed in the corresponding deletion strains (Δ*hmg1*, Δ*hmg2*, Δ*erg2*, Δ*erg3*, Δ*erg4*, Δ*erg5*, Δ*erg6*, Δ*erg24*, and Δ*erg28*). The complementation studies were performed in glucose media (Glu-media), which allows a low level of gene expression from the leaky *GAL1* plasmid promoter as opposed to the full induction and overexpression that would be induced from galactose media (Gal-media). MICs to the antifungals cycloheximide (CHX), fluconazole (FLC), lovastatin (LOV), fenpropimorph (FEN), and nystatin (NYS) were performed on the strains to test complementation (Table S1).

***HMG1*:** Deletion strain Δ*hmg1* is resistant to FLC and hyper-susceptible to LOV compared to the WT strain. The FLC resistant phenotype was reversed and restored to the WT susceptible level and the LOV hyper-susceptibility was restored to WT LOV susceptibility in the presence of the plasmids expressing *HMG*1.

***HMG2*:** Deletion strain Δ*hmg2* showed no phenotypic changes from WT for the conditions tested and so the corresponding plasmid expression in the presence of glucose media could not be used to show complementation in the Δ*hmg2* strain*.* However, the plasmid carrying *HMG2* did complement the FLC resistance phenotype of Δ*hmg1* restoring the WT FLC susceptibility when expressed in the Δ*hmg1* strain.

***ERG24*:** Deletion strain Δ*erg24* showed no phenotypic changes from WT for the conditions tested and so the corresponding plasmid expression in the presence of glucose media could not be used to show complementation. However, overexpression of *ERG24* in the Δ*erg24* strain using galactose media (Gal-media) as opposed to Glu-media provided resistance to FEN.

***ERG28*:** Deletion strain Δ*erg28* showed no phenotypic changes from WT for the conditions tested and so the corresponding plasmid expression in the presence of glucose media could not be used to show complementation.

***ERG6*:** Strain Δ*erg6* is resistant to FLC and NYS compared to the WT strain. The FLC and NYS resistant phenotype was reversed and restored to the WT susceptible levels in the presence of the plasmid expressing *ERG6*. Hyper-susceptibility to LOV and FEN was observed in the Δ*erg6* strain. The plasmid expressing *ERG6* in the Δ*erg6* strain restored LOV and FEN susceptibility to the WT level.

***ERG2*:** FLC hyper-susceptibility was observed in the Δ*erg2* strain and was restored to WT FLC susceptibility in the presence of the plasmid expressing *ERG2*. In addition, a slow growth rate observed in the Δ*erg2* strain was restored to WT growth in the presence of the plasmid expressing *ERG2*.

***ERG3*:** Strain Δ*erg3* is resistant to FLC and NYS compared to the WT strain. The FLC and NYS resistant phenotype was reversed and restored to the WT susceptible levels in the presence of the plasmid expressing *ERG*3. FEN hyper-susceptibility was observed in the Δ*erg3* strain and the plasmid expressing *ERG3* restored FEN susceptibility to the WT level.

***ERG5*:** NYS resistance was observed in the Δ*erg5* strain and was restored to the WT NYS susceptible level in the presence of the plasmid expressing *ERG5*.

***ERG4*:** NYS and FEN resistance was observed in the Δ*erg4* strain and was restored to the WT NYS susceptible level in the presence of the plasmid expressing *ERG4*.

Complementation analyses have demonstrated that the plasmids carrying eight of the nine non-essential genes (*HMG1*, *HMG2*, *ERG2*, *ERG3*, *ERG4*, *ERG5*, *ERG6* and *ERG24*) are functional as shown in Table S2. The complementation of Δ*erg28* with the plasmid carrying *ERG28* did not show a phenotype.
