# Supplementary material for: Overexpression or Deletion of Ergosterol Biosynthesis Genes Alters Doubling Time, Response to Stress Agents, and Drug Susceptibility in Saccharomyces cerevisiae
Source: mBio. 2018 Jul 24;9(4):e01291-18. doi: 10.1128/mBio.01291-18 (PMC6058291; doi:10.1128/mBio.01291-18)
Supplement: TABLE S3 [file mbo004183972st3.docx]

**Supplemental Table 3**. Oligos Used for ERG Gene Amplification from Genomic DNA

|  | **FORWARD OLIGO 5’ - 3’** | **REVERSE OLIGO 5’ - 3’** |
| --- | --- | --- |
| ***ERG1*** | CCTCTATACTTTAACGTCAAGGAGAAAAAACCTATAATGTCTGCTGTTAACGTTGC | TGAATGTAAGCGTGACATAACTAATTACATGATTTAACCAATCAACTCACCAAA |
| ***ERG7*** | CCTCTATACTTTAACGTCAAGGAGAAAAAACCTATAATGACAGAATTTTATTCTGA | TGAATGTAAGCGTGACATAACTAATTACATGATTTAAAGCGTATGTGTTTCAT |
| ***ERG11*** | CCTCTATACTTTAACGTCAAGGAGAAAAAACCTATAATGTCTGCTACCAAGTCAAT | TGAATGTAAGCGTGACATAACTAATTACATGATTTAGATCTTTTGTTCTGGAT |
| ***ERG24*** | CCTCTATACTTTAACGTCAAGGAGAAAAAACCTATAATGGTATCAGCTTTGAATCC | TGAATGTAAGCGTGACATAACTAATTACATGATTTAATAAACATATGGAATGA |
| ***ERG3*** | CCTCTATACTTTAACGTCAAGGAGAAAAAACCTATAATGGATTTGGTCTTAGAAGT | TGAATGTAAGCGTGACATAACTAATTACATGATTCAGTTGTTCTTCTTGGTAT |
| ***ERG6*** | CCTCTATACTTTAACGTCAAGGAGAAAAAACCTATAATGAGTGAAACAGAATTGAG | TGAATGTAAGCGTGACATAACTAATTACATGATTTATTGAGTTGCTTCTTGGG |
| ***ERG9*** | CCTCTATACTTTAACGTCAAGGAGAAAAAACCTATAATGGGAAAGCTATTACAATT | TGAATGTAAGCGTGACATAACTAATTACATGATTCACGCTCTGTGTAAAGTGT |
| ***HMG1*** | CCTCTATACTTTAACGTCAAGGAGAAAAAACCTATAATGCCGCCGCTATTCAAGGG | TGAATGTAAGCGTGACATAACTAATTACATGATTTAGGATTTAATGCAGGTGA |
| ***ERG26*** | CCTCTATACTTTAACGTCAAGGAGAAAAAACCTATAATGTCAAAGATAGATTCAGT | TGAATGTAAGCGTGACATAACTAATTACATGATTTACAAACCTTCGTCCATCCAGGCC |
| ***ERG4*** | CCTCTATACTTTAACGTCAAGGAGAAAAAACCTATAATGGCAAAGGATAATAGTGA | TGAATGTAAGCGTGACATAACTAATTACATGATCTAGAAAACATAAGGAATAAAGACG |
| ***ERG5*** | CCTCTATACTTTAACGTCAAGGAGAAAAAACCTATAATGAGTTCTGTCGCAGAAAA | TGAATGTAAGCGTGACATAACTAATTACATGATTTATTCGAAGACTTCTCCAG |
| ***ERG25*** | CCTCTATACTTTAACGTCAAGGAGAAAAAACCTATAATGTCTGCCGTTTTCAACAA | TGAATGTAAGCGTGACATAACTAATTACATGATTTAGTTAGTCTTCTTTTGAG |
| ***ERG27*** | CCTCTATACTTTAACGTCAAGGAGAAAAAACCTATAATGAACAGGAAAGTAGCTAT | TGAATGTAAGCGTGACATAACTAATTACATGATTTAAATGGGGGTTCTAGTTT |
| ***ERG2*** | CCTCTATACTTTAACGTCAAGGAGAAAAAACCTATAATGAAGTTTTTCCCACTCCT | TGAATGTAAGCGTGACATAACTAATTACATGATTTAGAACTTTTTGTTTTGC |
| ***ERG8*** | CCTCTATACTTTAACGTCAAGGAGAAAAAACCTATAATGTCAGAGTTGAGAGCCTT | TGAATGTAAGCGTGACATAACTAATTACATGATTTATTTATCAAGATAAGTTTCC |
| ***ERG10*** | CCTCTATACTTTAACGTCAAGGAGAAAAAACCTATAATGTCTCAGAACGTTTACAT | TGAATGTAAGCGTGACATAACTAATTACATGATTCATATCTTTTCAATGACAA |
| ***ERG13*** | CCTCTATACTTTAACGTCAAGGAGAAAAAACCTATAATGAAACTCTCAACTAAACT | TGAATGTAAGCGTGACATAACTAATTACATGATTTATTTTTTAACATCGTAAG |
| ***ERG12*** | CCTCTATACTTTAACGTCAAGGAGAAAAAACCTATAATGTCATTACCGTTCTTAAC | TGAATGTAAGCGTGACATAACTAATTACATGATTTATGAAGTCCATGGTAAAT |
| ***ERG19*** | CCTCTATACTTTAACGTCAAGGAGAAAAAACCTATAATGACCGTTTACACAGCATC | TGAATGTAAGCGTGACATAACTAATTACATGATTTATTCCTTTGGTAGACCAG |
| ***IDI1*** | CCTCTATACTTTAACGTCAAGGAGAAAAAACCTATAATGACTGCCGACAACAATAG | TGAATGTAAGCGTGACATAACTAATTACATGATTTATAGCATTCTATGAATTTGCC |
| ***ERG20*** | CCTCTATACTTTAACGTCAAGGAGAAAAAACCTATAATGGCTTCAGAAAAAGAAATTAGGAGAGAG | TGAATGTAAGCGTGACATAACTAATTACATGATCTATTTGCTTCTCTTGTAAACTTTGTTC |
| ***HMG2*** | CCTCTATACTTTAACGTCAAGGAGAAAAAACCTATAATGTCACTTCCCTTAAAAAC | TGAATGTAAGCGTGACATAACTAATTACATGATTTATAATAATGCTGAGGTTTTAC |
| ***ERG28*** | CCTCTATACTTTAACGTCAAGGAGAAAAAACCTATAATGTTCAGCCTACAAGACGT | TGAATGTAAGCGTGACATAACTAATTACATGATTTACCAAGCAACACCAGTGT |
| ***ERG29*** | CCTCTATACTTTAACGTCAAGGAGAAAAAACCTATAATGTCGTTAAAGGATAGGTA | TGAATGTAAGCGTGACATAACTAATTACATGATTCATAGTATGAATTCGCATT |
| ***NCP1*** | CCTCTATACTTTAACGTCAAGGAGAAAAAACCTA TAATGCCGTTTGGAATAGACAA | TGAATGTAAGCGTGACATAACTAATTACATGATTTACCAGACATCTTCTTGGT |
