# Supplementary material for: Overexpression or Deletion of Ergosterol Biosynthesis Genes Alters Doubling Time, Response to Stress Agents, and Drug Susceptibility in Saccharomyces cerevisiae
Source: mBio. 2018 Jul 24;9(4):e01291-18. doi: 10.1128/mBio.01291-18 (PMC6058291; doi:10.1128/mBio.01291-18)
Supplement: TABLE S4 [file mbo004183972st4.docx]

**Supplemental Table 4**. Oligos used for ERG Gene Sequencing and qRT-PCR

| **Oligo Name** | **Sequence (5’-3’)** | **Used for** |
| --- | --- | --- |
| Sc-ACT1-F-qrt | ATGGATTCTGAGGTTGCTGC | qRT-PCR |
| Sc-ACT1-R-qrt | GGTGTCTTGGTCTACCGACG | qRT-PCR |
| Sc-ERG1-F-qrt | GGAATTCAAAGCCCACTTGA | qRT-PCR+sequencing |
| Sc-ERG1-R-qrt | TAACGTGACCGTGCATAGGA | qRT-PCR+sequencing |
| Sc-ERG3-F-qrt | ATCCCATGGATGTCGATGTT | qRT-PCR+sequencing |
| Sc-ERG3-R-qrt | TTGTGCAGAGCACGGTAGAC | qRT-PCR+sequencing |
| Sc-ERG4-F-qrt | CGGGTTTCCACTGCTAATGT | qRT-PCR+sequencing |
| Sc-ERG4-R-qrt | CCCGGGCAACGTATAGTAGA | qRT-PCR+sequencing |
| Sc-ERG6-F-qrt | CAACTCTGCCCAAAAGGAAG | qRT-PCR+sequencing |
| Sc-ERG6-R-qrt | CTATCGAGGCAGCGAAACTC | qRT-PCR+sequencing |
| Sc-ERG7-F-qrt | TCGATGCGTGTCATAATGGT | qRT-PCR+sequencing |
| Sc-ERG7-R-qrt | CACGGTGGATTTGTCAACAG | qRT-PCR+sequencing |
| Sc-ERG7-Fa | TTCCATGGTCCACAGGGTAT | sequencing |
| Sc-ERG7-Rb | TTTTGTTGAGAAGCCCCAAG | sequencing |
| Sc-ERG9-F-qrt | TGAAAGCATGGGTCTTTTCC | qRT-PCR+sequencing |
| Sc-ERG9-R-qrt | CAACCCCAGTTGTTCGTTTT | qRT-PCR+sequencing |
| Sc-ERG11-F-qrt | TTGTTGCACTTGGCTGAAAG | qRT-PCR+sequencing |
| Sc-ERG11-R-qrt | CGGTGAATGTTGAATTGGTG | qRT-PCR+sequencing |
| Sc-ERG24-F-qrt | GGCAGTTTTGGACGTCATTT | qRT-PCR+sequencing |
| Sc-ERG24-R-qrt | TGAAGCTGGCAACATAGCAG | qRT-PCR+sequencing |
| Sc-ERG26-F-qrt | GCCCAGGTGATAGGCAATTA | qRT-PCR+sequencing |
| Sc-ERG26-R-qrt | TCTGCCTTCCACACAGTACG | qRT-PCR+sequencing |
| Sc-HMG1-F-qrt | GCCATTCGTGATAGGTTCGT | qRT-PCR+sequencing |
| Sc-HMG1-R-qrt | CTCGATTGCGCAGATGATAA | qRT-PCR+sequencing |
| Sc-HMG1-F-qrt_a | GTCCGTTTCCCAACTTTGAA | sequencing |
| Sc-HMG1-R-qrt_b | CGACGACACTCTTACCACGA | sequencing |
| Sc-YEF3-F-qrt | GCAACACGCTTTTGCTCATA | qRT-PCR |
| Sc-YEF3-R-qrt | GGACATCATTGGAACCCATC | qRT-PCR |
| Sc-UPC2-F-qrt | GCGTTCAGGAAGAAAACAGC | qRT-PCR |
| Sc-UPC2-R-qrt | CTGCACGTTCATGATGCTCT | qRT-PCR |
| Sc-ERG5-F-qrt | CCCCTAACTATACCGCACCA | qRT-PCR+sequencing |
| Sc-ERG5-R-qrt | GTGGACCACAACCAAAAACC | qRT-PCR+sequencing |
| Sc-ERG25-F-qrt | CCGTTGGTATGCCAATTCTT | qRT-PCR+sequencing |
| Sc-ERG25-R-qrt | CCCACCATCTGAAAGAGGAA | qRT-PCR+sequencing |
| Sc-ERG27-F-qrt | GCCGTGTAGAGGATTTGGAA | qRT-PCR+sequencing |
| Sc-ERG27-R-qrt | GACGCCCACCAGTTGTATCT | qRT-PCR+sequencing |
| Sc-ERG2-F-qrt | AGACGCACTTGCCTCTCATT | qRT-PCR+sequencing |
| Sc-ERG2-R-qrt | TCGTCAGCAAAGTGAACACC | qRT-PCR+sequencing |
| Sc-ERG8-F-qrt | TGCTCATTGTCAAGCTCAGG | qRT-PCR+sequencing |
| Sc-ERG8-R-qrt | AACCAAATGCGCCAGTTTAC | qRT-PCR+sequencing |
| Sc-ERG10-F-qrt | GTTACTGCCGCTAACGCTTC | qRT-PCR+sequencing |
| Sc-ERG10-R-qrt | AAGCCTTTGGAACTGCAAGA | qRT-PCR+sequencing |
| Sc-ERG13-F-qrt | GGCAAGCGTGTTGGTTTATT | qRT-PCR+sequencing |
| Sc-ERG13-R-qrt | GGCAGCTTCGTAATCCTTTG | qRT-PCR+sequencing |
| Sc-ERG12-F-qrt | TGGACTGCTTGTCTCAATCG | qRT-PCR+sequencing |
| Sc-ERG12-R-qrt | CAGTCCCACCCAAGTCTGTT | qRT-PCR+sequencing |
| Sc-ERG19-F-qrt | GACTGGCCTCAGATGAAAGC | qRT-PCR+sequencing |
| Sc-ERG19-R-qrt | CAAACATGTGGCATGGAAAG | qRT-PCR+sequencing |
| Sc-IDI1-F-qrt | TGCAAAACCAAACACCTGAA | qRT-PCR+sequencing |
| Sc-IDI1-R-qrt | ATCGTCCCAATCCAAAACAA | qRT-PCR+sequencing |
| Sc-ERG20-F-qrt | TCTACTTGCCTGTCGCATTG | qRT-PCR+sequencing |
| Sc-ERG20-R-qrt | GCAAGTTCCAATGCCTTGTT | qRT-PCR+sequencing |
| Sc-HMG2-F-qrt | TTCGGGACCAATTTATCAGC | qRT-PCR+sequencing |
| Sc-HMG2-R-qrt | GCCAGAAGAAGCAGGCATAC | qRT-PCR+sequencing |
| Sc-HMG2-F-qrt_a | TGACTAGAGGCCCAGTCGTT | sequencing |
| Sc-HMG2-R-qrt_b | TAAAAAGCAAATCGCCTGCT | sequencing |
| Sc-ERG28-qrt_F | CCCACTGAAACAACCCATTT | qRT-PCR+sequencing |
| Sc-ERG28-qrt-R | GAAGTGGAATAGGGCAACCA | qRT-PCR+sequencing |
| Sc-ERG29-qrt_F | TTGCGTGTATTGAGGCTGAG | qRT-PCR+sequencing |
| Sc-ERG29-qrt_R | TCCCCGTATCAATCTTCAGG | qRT-PCR+sequencing |
| Sc-Ncp1-qrt_F | TTCAACCTGGACCCTGAAAC | qRT-PCR+sequencing |
| Sc-Ncp1-qrt_R | GACATGGACGGTTTGCTTTT | qRT-PCR+sequencing |
| GAL-SC-Seq-FWD | CTGGGGTAATTAATCAGCGA | sequencing |
| GAL-SC-Seq-REV | AGGGCGTGAATGTAAGCGTG | sequencing |
